# A Practical Approach to New (5*Z*) 2-Alkylthio-5-arylmethylene-1-methyl-1,5-dihydro-4*H*-imidazol-4-one Derivatives

**DOI:** 10.3390/molecules16097377

**Published:** 2011-08-30

**Authors:** Khadidja Bourahla, Ludovic Paquin, Olivier Lozach, Laurent Meijer, François Carreaux, Jean Pierre Bazureau

**Affiliations:** 1 Chemical Engineering & Molecules for Life Sciences Group, Université de Rennes 1, Sciences Chimiques de Rennes (SCR), UMR CNRS 6226, Bât. 10A, Campus de Beaulieu, Avenue du Général Leclerc, CS 74205, 35042 Rennes Cedex, France; 2 Protein Phosphorylation and Human Disease Group, Station Biologique CNRS, Place G. Tessier, BP 74, 29682 Roscoff, France

**Keywords:** 1,5-dihydro-4*H*-imidazol-4one, Knoevenagel condensation, microwave irradiation, S-alkylation

## Abstract

A practical protocol for the preparation of (5*Z*)-2-alkylthio-5-arylmethylene-1-methyl-1,5-dihydro-4*H*-imidazol-4-one derivatives is reported. The new compounds were obtained in good yield and stereoselectivity in two steps, namely a solvent-free Knoevenagel condensation under microwave irradiation, followed by an S-alkylation reaction with various halogenoalkanes.

## 1. Introduction

**Figure 1:**
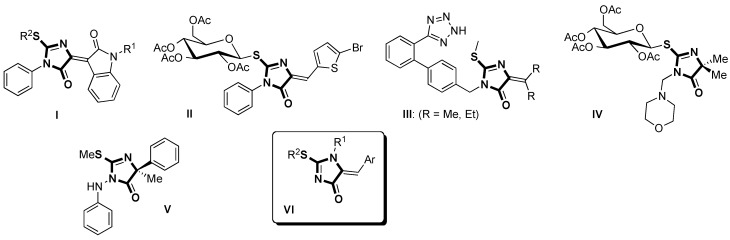
Select 3,5-dihydro-4*H*-imidazol-4-one derivatives with biological activities.

2-Thiohydantoins (4-oxoimidazolidine-2-thiones) [1,2] and their 2-alkylthio-3,5-dihydro-4*H*-imidazol-4-one derivatives are a biologically important class of compounds in the fields of drugs, pharmaceutical intermediates and agrochemicals. As examples, the isatinylidene derivative **I** (Figure 1) exhibits immunosuppressive activity [3] and the thioglycosyl hydantoin [4] **II** possesses a broad spectrum antitumor activity against a wide range of different human cell lines from nine tumor subpanels causing both cytostatic and cytotoxic effects. The 5-arylmethylene-2-methylthio-imidazol-4-ones **III** substituted with a biphenyltetrazole (BTP) group at the C-2 position show activities as angiotensin II receptor antagonists [5] and the 3-morpholinomethyl-5,5-dimethyl-2-thioglycosyl-imidazol-4-one **IV** has been also identified as a potential AZT analogue [6]. The 2-thiohydantoin derivatives have not only been used in medicinal chemistry, but have also been developed as fungicides [7] [e.g., fenamidone (**V)** [8,9]] and herbicides [10]. Recent work [11] by Wang's group has established that esters of 5-(4-hydroxybenzyl)thiohydantoins exhibit good herbicidal activity against *Zea mays* and *Arabidopsis thaliana*.

On the other hand, the synthesis and biological properties of 2-alkylthio-5-arylmethylene-1,5-dihydro-4*H*-imidazol-4-one derivatives **VI** have rarely been the subject of detailed investigations reported in the literature. A 17-year-old study of Unangst and co-workers only reported the preparation of (*5Z*)-[[3,5-bis(1,1-dimethylethyl)-4-hydroxyphenyl]methylene]-1,5-dihydro-1-methyl-2-methylthio-4*H*-imidazol-4-one for an inflammation therapy program [12]. In 1993, a report described the biological activity of the same compound as a potent antiviral agent for the human immunodeficiency virus (HIV) [13]. More recently, the use of 5-arylidene-1-methyl-1,5-dihydro-4*H*-imidazol-4-one as a convenient synthetic intermediate in a sulfur/nitrogen displacement has been studied with one example [14].

Due to the biological activity associated with the imidazolone moiety, we embarked on a project to investigate possible bioactive molecules based on 2-alkylthio-5-arylmethylene-1-methyl-1,5-dihydro-4*H*-imidazol-4-one derivatives of the imidazolone core. Herein, we report our results concerning the synthesis of these new 2-alkylthio-5-arylmethylene derivatives based on the 1-methyl-2-thiohydantoin scaffold and their biological evaluation as protein kinase inhibitors. The protein kinases of the human kinome represent a wide family of disease relevant targets for identification, preparation and optimization of potential therapeutic agents in structure-activity relationship (SAR) studies [15].

## 2. Results and Discussion

Most of the 2-alkylthio-5-arylmethylene imidazol-4-ones syntheses described in the literature involve: (i) a Knoevenagel condensation of an aryl/heteroaryl aldehyde with a 2-thiohydantoin followed by a regioselective *S*-alkylation [16] or (ii) reaction of vinylisothiocyanate [17] (obtained from an iminophosphorane [18] and carbon disulfide) with a primary amine giving the 5-arylmethylene-2-thioxo-imidazol-4-one structure [19,20], which is then converted into the *S*-alkyl derivative by the action of an alkylating reagent.

**Figure 2:**
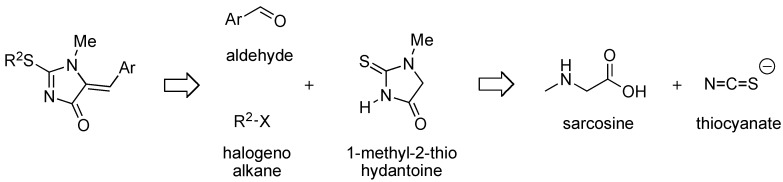
Components used for the synthesis of 2-alkylthio-5-arylmethylene-1-methyl-1,5-dihydro-4*H*-imidazol-4-ones.

For this project, the needed 2-alkylthio-5-arylmethylene-1-methyl-1,5-dihydro-4*H*-imidazol-4-one can be built from sarcosine and a thiocyanate as precursors of the 1-methyl-2-thiohydantoin scaffold, aldehydes and halogenoalkanes, to introduce diversity at the C-5 position and on the sulfur atom at the C-2 position respectively, by Knoevenagel condensation and *S*-alkylation reactions (Figure 2).

**Scheme 1:**
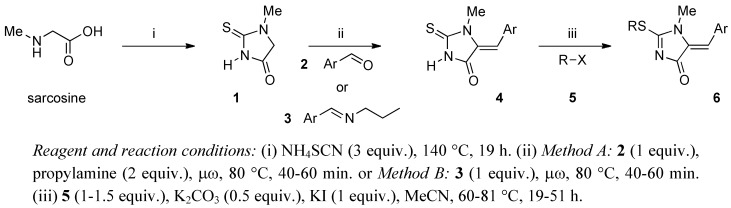
Route used for the synthesis of 2-alkylthio-5-arylmethylene-1-methyl-1,5-dihydro-4*H*-imidazol-4-ones.

As illustrated in Scheme 1, the synthesis started with the preparation of 1-methyl-2-thiohydantoin (**1**) from commercially available sarcosine and ammonium thiocyanate. 

Complete conversion according to the modified procedure of Kenyon *et al.* [21,22] was observed after 19 hours at 140 °C, affording the desired starting compound **1** in 90% yield. In the second step, for the Knoevenagel condensations from aryl aldehydes and thiohydantoins, several methods have been employed in the literature. Many of these methods suffer from one or more limitations such as requiring harsh reaction conditions, producing low to moderate yields, relatively long reaction times and cumbersome experimental processes. Among these reported methods, the Knoevenagel reaction has been performed in the presence of mineral or organic bases with various solvents: ethanolamine in absolute ethanol [23], potassium hydroxide in anhydrous ethanol [24], sodium hydride in anhydrous acetonitrile [25]. The utility of microwave irradiation (μω) to carry out organic reaction has now become a regular feature. The main benefits of performing the reaction under microwave conditions are the higher product yields and the significant rate-enhancements that can be observed. It’s clear that application of microwave technology to the rapid synthesis of potential biological molecules is a useful tool for the medicinal chemistry community, for whom reaction speed is of great importance [26,27]. Moreover, when a reaction is carried out in a microwave reactor, the use of solvent can be avoided [28,29], allowing eco-friendly synthesis and offering several advantages, such as reduced risk of explosions and easier work-up. In this context, we have examined two experimental protocols for the synthesis of 5-arylmethylene-1-methyl-2-thiohydantoins **4**.

The results of the two methods investigated for the preparation of Knoevenagel products **4** are presented in Table 1. In Method A, 1-methyl-2-thiohydantoin (**1**) was coupled with commercial aryl aldehydes **2(a-e)** in the presence of two equivalents of propylamine at 80 °C under microwave irradiation (in a Synthewave® 402 reactor [30]) for a reaction time ranging from 40 to 60 minutes. In Method B, an equimolecular mixture of the starting hydantoin **1** and arylaldimine **3** was heated at 80 °C under microwave irradiation for the same reaction time. The arylaldimines **3** were prepared in good yields according to a solvent-free microwave protocol developed in our laboratory [31]. The reactions of both methods were conveniently monitored by ^1^H- NMR or by TLC on precoated plates of silica gel with an appropriate eluant. As can be seen from inspection of the data presented in Table 1, the 5-arylmethylene-1-methyl- 2-thioxo-imidazolidin-4-ones **4(a-e)** were prepared in better yields (86-98%) using the microwave irradiation reaction conditions (Method A). It is noteworthy that for safety reasons, a 4-min. heating ramp was performed before the temperature was maintained at the selected maximum of 80 °C (power 80 W). The structure of the new compounds **4(a-e)** were substantiated by ^1^H-, ^13^C-NMR and HRMS. In all cases, compounds **4** were obtained in a stereospecific way and the geometry of the double bond was attributed as being *Z* by the shielding effect of the carbonyl group C-4 on the olefinic proton H-5 (δ_H-5_ = 6.34-6.85 ppm) [32,33].

**Table 1:** Results for the solvent-less preparation of 5-arylmethylene-1-methyl-2-thioxo imidazolin-4-ones **4(a-e)** under microwave from aldehydes **2(a-e)** (Method A) or aldimines **3(a-e)** (Method B).

Product	Method A		Method B	
	Starting reagent	Yield *^a^* of 4 (%)	Starting reagent	Yield *^a^* of 4 (%)
**4a**	**2a**	96	**3a**	88
**4b**	**2b**	96	**3b**	92
**4c**	**2c**	90	**3c**	90
**4d**	**2d**	86	**3d**	-^b^
**4e**	**2e**	98	**3e**	92
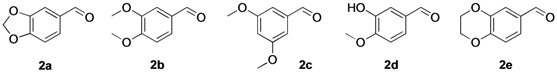				

*^a^* Yield of isolated product after purification by recrystallization; *^b^* The reaction failed under microwave and also in an oil bath using the same reaction conditions.

With the 5-arylmethylene-1-methyl-2-thioxo imidazolin-4-one derivatives **4** in hand, we designed an experimental strategy for the preparation of the 2-alkylthio-1,5-dihydro-4*H*-imidazol-4-ones **6**. For this study, a set of different halogeno compounds **5** represents the second point of diversity in this scaffold by using commercial products [ethyl iodide (**5a**), allyl bromide (**5b**), propyl bromide (**5c**), chloroacetonitrile (**5d**), ethyl bromoacetate (**5e**) and 3-bromopropanol (**5f**)]. Owing to the lesser reactivity of the chloro and bromo alkanes **5(b-f)**, one equivalent of potassium iodide was added in the reaction mixtures and the reagents were covered with dry acetonitrile. After work-up (elimination of the salts and solvent), all the crude products were purified easily by recrystallization in ethanol. The reaction conditions listed in Table 2 showed that the *S*-alkylations could be carried out at various reaction temperatures (60-81 °C) with a reaction time ranging from 14 to 51 hours. As seen from the results, it can be observed that these *S*-alkylations gave moderate to good yields (42-68%). The structural assignment of the new 2-alkylthio-5-arylmethylene-1-methyl 1,5-dihydro-4*H*-imidazol-4-ones **6(a-g)** is based on spectroscopic data (^1^H-, ^13^C-NMR, HRMS). It should be noted that this alkylation step gave regioselective *S*-alkylation with retention of the (*5Z*)-stereochemistry (δ_H-5_ = 6.83-7.21 ppm).

The (5*Z*) 2-alkylthio-5-arylmethylene-1-methyl-1,5-dihydro-4*H*-imidazol-4-ones **6(a-g)** were next tested for their potential inhibitory action on two protein kinases, CDK1/cyclin B and GSK-3α/β. Kinases were purified and essayed in the presence of 15 mM ATP and appropriate protein substrates (histone H1 for CDK1/cyclin B, GS-1 peptide for GSK-3α/β) as previously described [34,35,36]. Investigation of *in-vitro* bioactivity revealed that none of the compounds **6(a-g)** was active (IC_50_ > 10 μM).

**Table 2:** Results for the preparation of (*5Z*)-2-alkylthio-5-arylmethylene-1-methyl 1,5-dihydro-4*H*-imidazol-4-one **6(a-e)** from (*5Z*)-5-arylmethylene-1-methyl-2-thioxo imidazol-in-4-ones **4** and halogeno alkanes **5**.

Product 6	R	Starting product 4	Reagent 5	Reaction conditions: temperature *^a^* and reaction time	Yield *^b^* of 6 (%)
**6a**	CH_3_CH_2_	**4a**	**5a**	60 °C, 51 h	60
**6b**	CH_2_=CH-CH_2_	**4a**	**5b**	66 °C, 48 h	65
**6c**	CH_3_CH_2_CH_2_	**4a**	**5c**	65 °C, 24 h	62
**6d**	NC-CH_2_	**4a**	**5d**	81 °C, 19h	66
**6e**	EtO_2_C-CH_2_	**4a**	**5e**	60 °C, 14 h	68
**6f**	EtO_2_C-CH_2_	**4b**	**5e**	60 °C, 19 h	62
**6g**	HOCH_2_CH_2_CH_2_	**4a**	**5f**	70 °C, 46 h	42
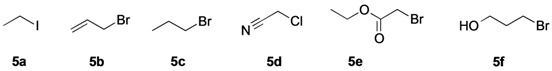					

*^a^* Reactions were run in a thermostated oil bath, temperature variation ±1 °C; *^b^* Yield of isolated product after purification by recrystallization.

## 3. Experimental

### 3.1. General

Melting points were determined on a Kofler melting point apparatus and are uncorrected. Thin-layer chromatography (TLC) was accomplished on 0.2-mm precoated plates of silica gel 60 F-254 (Merck) and visualization was made with ultraviolet light (254 and 312 nm) or with a fluorescent indicator. ^1^H- and ^13^C-NMR spectra were recorded on a Bruker AC 300 P spectrometer at 300 MHz and 75 MHz, respectively. Chemical shifts are expressed in parts per million downfield from tetramethylsilane as an internal standard. Data are given in the following order: d value, multiplicity (s, singlet; d, doublet; t, triplet; q, quartet; m, multiplet; br, broad), number of protons, coupling constants *J* is given in Hertz. The mass spectra (HRMS) were taken respectively on a Varian MAT 311 at an ionizing potential of 70 eV in the Centre Régional de Mesures Physiques de l’Ouest (CRMPO, Rennes, France). Reactions under microwave irradiations were realized in the Synthewave® 402 apparatus (Merck Eurolab, Div. Prolabo, France). The microwave instrument consists of a continuous focused microwave power output from 0 to 300W. All the experiments were performed using stirring option. The target temperature was reached with a ramp of 4 minutes and the chosen microwave power stay constant to hold the mixture at this temperature. The reaction temperature is monitored using calibrated infrared sensor and the reaction time includes the ramp period. Acetonitrile was distilled over calcium chloride after standing overnight and stored over molecular sieves (3Å). Solvents were evaporated with a Büchi rotary evaporator. All reagents were purchased from Acros, Aldrich Chimie, Fluka France and used without further purification.

*1-Methyl-2-thioxo imidazolidin-4-one* (**1**): This starting compound was prepared in a 50 mL two-necked round-bottomed flask, equipped with a magnetic stirrer and reflux condenser by the fusion of commercial sarcosine (4 g, 44.9 mmol) with ammonium thiocyanate NH_4_SCN (10.26 g, 134.7 mmol, 3 equiv.) at 140 °C under a slow stream of nitrogen. After 19 hr of heating under vigorous stirring, the dark red solution was cooled. The solid cake which formed was broken up and washed with 20 mL of water onto a filter. The crystals were then washed successively with deionized water (three 15 mL portions), 95% ethanol (one 20 mL portion) and hexane (one 20 mL portion). The precipitated product was further dried under high vacuum (10^−2^ Torr) at 30 °C for 2 hours, to give the desired 1-methyl-2-thioxoimidazolidin-4-one (**1**) as a powder that was used without further purification. Yield = 90%. Mp = 230-232 °C. ^1^H-NMR [(CD_3_)_2_SO] δ = 3.12 (s, 3H, NCH_3_); 4.20 (s, 2H, CH_2_); 11.67 (br s, 1H, NH). ^13^C-NMR [(CD_3_)_2_SO] δ = 33.3 (NCH_3_); 55.7 (CH_2_, C-5); 172.7 (C=O, C-4); 182.0 (C=S, C-2). HRMS, *m/z* found: 130.0207 (calculated for C_4_H_6_N_2_OS, M^+^. requires: 130.0201).

### 3.2. General Procedure for the Solventless Synthesis of (5Z) 5-Arylmethylene-1-methyl-2-thioxo-imidazolin-4-ones ***4*** under Microwave Dielectric Heating according to Method A and Method B:

*Method A*: A mixture of 1-methyl-2-thioxo-imidazolidin-4-one (**1**, 1.3 g, 10 mmol), propylamine (1.64 mL, 20 mmol, 2 equiv.) and appropriate aromatic aldehyde **2** (10 mmol) was placed in a cylindrical quartz reactor (Ø = 2.8 cm). The reactor was then introduced into a Synthewave® 402 Prolabo microwave reactor (P = 300 Watt). The stirred mixture was irradiated at 80 °C (with a power of 90 Watt) for 40-60 min. After microwave dielectric heating, the crude reaction mixture was allowed to cool down at room temperature and was concentrated by rotary evaporation under reduced pressure. Ethanol (10 mL) was added directly in the crude reaction mixture and the resulting precipitated crude product **4** was filtered off and purified by recrystallization from ethanol to give the desired *(5Z)* 5-arylmethylene-1-methyl-2-thioxo-imidazolin-4-one **4**.

*Method B:* A mixture of 1-methyl-2-thioxo-imidazolidin-4-one (**1**, 1.3 g, 10 mmol) and an appropriate aldimine **3** (10 mmol) was placed in a cylindrical quartz reactor (Ø = 2.8 cm). The reactor was then introduced into a Synthewave® 402 Prolabo microwave reactor (P = 300 Watt). The stirred mixture was irradiated at 80 °C (with a power of 90 Watt) for 40-60 min. After microwave dielectric heating, the crude reaction mixture was allowed to cool down at room temperature and was concentrated by rotary evaporation under reduced pressure. Ethanol (10 mL) was added directly in the crude reaction mixture and the resulting precipitated crude product **4** was filtered off and purified by recrystallization from ethanol to give the desired *(5Z*) 5-arylmethylene-1-methyl-2-thioxo-imidazolin-4-one **4**.

*(5Z)-5-(1,3-Benzodioxol-5-ylmethylene)-1-methyl-2-thioxoimidazolidin-4-one* (**4a**): *Method A:* The product **4a** was prepared from **1** (1.3 g, 10 mmol), propylamine (1.64 mL, 20 mmol, 2 equiv.) and piperonal (**2a**, 1.502 g, 10 mmol) with a reaction of 50 min. Yield = 96%.

*Method B*: The product **4a** was prepared from **1** (650 mg, 5 mmol) and *N*-(1,3-benzodioxol-5-ylmethylene)propan-1-amine (**3a**, 630 mg, 5 mmol) with a reaction time 60 min. Yield = 88%.

Yellow needles, mp = 252-254 °C. ^1^H-NMR [(CD_3_)_2_SO] δ = 3.18 (s, 3H, NCH_3_); 6.09 (s, 2H, OCH_2_O); 6.54 (s, 1H, C=CH); 6.96 (d, 1H, *J* = 8.1 Hz, H-5', Ar); 7.27 (d, 1H, *J* = 8.1 Hz, H-6', Ar); 7.45 (s, 1H, H-2', Ar); 12.22 (br s, 1H, NH). ^13^C-NMR [(CD_3_)_2_SO] δ = 27.6 (NCH_3_); 102.10 (OCH_2_O); 109.10 (C-5); 109.81 (C-2); 113.70 (C=CH); 125.11 (C=CH); 126.90 (C-6); 126.91 (C-1); 148.40 (C-4); 149.02 (C-3); 164.61 (C=O); 179.0 (C=S). HRMS, *m/z* found: 262.0409 (calculated for C_12_H_10_N_2_O_3_S, M^+^. requires: 262.0412).

*(5Z)-5-(3,4-Dimethoxybenzylidene)-1-methyl-2-thioxoimidazolidin-4-one* (**4b**): *Method A*: The product **4b** was prepared from **1** (1.3 g, 10 mmol), propylamine (1.64 mL, 20 mmol, 2 equiv.) and 3,4-dimethoxybenzaldehyde (**2b**, 1.670 g, 10 mmol) with a reaction time of 40 min. Yield= 96%.

*Method B:* The product **4b** was prepared from **1** (1 g, 6.57 mmol) and *N*-[(3,4-dimethoxyphenyl)-methylene]propan-1-amine (**3b**, 1.12 mL, 13.14 mmol, 2 equiv.) with a reaction time 60 min. Yield = 92%.

Yellow needles, mp= 234-236 °C. ^1^H-NMR [(CD_3_)_2_SO] δ = 3.12 (s, 3H, NCH_3_); 3.92 (s, 3H, OCH_3_); 3.93 (s, 3H, OCH_3_); 6.44 (d, 1H, *J* = 8.8 Hz, H-5', Ar); 6.81 (s, 1H, C=CH); 7.24 (d, 1H, *J* = 6.4 Hz, H-6', Ar); 7.25 (s, 1H, H-2, Ar); 12.08 (br s, 1H, NH). ^13^C-NMR [(CD_3_)_2_SO] δ = 25.02 (NCH_3_); 55.51 (OCH_3_); 55.91 (OCH_3_); 113.82 (C-5); 114.53 (C-2); 116.20 (C=CH); 124.21 (C-6); 125.30 (C-1); 125.61 (C=CH); 148.30 (C-4); 149.11 (C-3); 166.32 (C=O); 178.80 (C=S). HRMS, *m/z* found: 278.0701 (calculated for C_13_H_14_N_2_O_3_S, M^+^. requires: 278.0725).

*(5Z)-5-(3,5-Dimethoxybenzylidene)-1-methyl-2-thioxoimidazolidin-4-one* (**4c**): *Method A*: The product **4c** was prepared from **1** (650 mg, 5 mmol), propylamine (1.32 mL, 10 mmol, 2 equiv.) and 3,5-dimethoxybenzaldehyde (**2c**, 1.240 g, 5 mmol) with a reaction of 40 min. Yield = 90%.

*Method B:* Compound **4c** was prepared from **1** (1 g, 6.57 mmol.) and *N*-[(3,5-dimethoxyphenyl)-methylene]propan-1-amine (**3c**, 1.12 mL, 13.14 mmol, 2 equiv.) with a reaction time 60 min. Yield = 90%.

Yellow needles, mp > 260 °C. ^1^H-NMR [(CD_3_)_2_SO] δ = 3.24 (s, 3H, NCH_3_); 3.83 (s, 6H, OCH_3_); 6.49 (s, 1H, H-4', Ar); 6.85 (s, 1H, C=CH); 7.40 (s, 2H, H-2', H-6', Ar); 12.02 (br s, 1H, NH). ^13^C- NMR [(CD_3_)_2_SO] δ = 25.21 (NCH_3_); 55.12 (OCH_3_); 103.04 (C-4); 109.31 (C-2); 123.52 (C=CH); 136.23 (C-1); 138.80 (C=CH); 160.62 (C-3); 167.31 (C=O); 178.84 (C=S). HRMS, *m/z* found: 278.0630 (calculated for C_13_H_14_N_2_O_3_S, M^+^. requires: 278.0725).

*(5Z)-5-(3-Hydroxy-4-methoxybenzylidene)-1-methyl-2-thioxoimidazolidin-4-one* (**4d**): The product **4d** was prepared from **1** (855 mg, 6.57 mmol), propylamine (1.08 mL, 13.14 mmol, 2 equiv.) and vanillin (**2d**, 1 g, 6.57 mmol) with a reaction of 60 min. according to the standard procedure for method A. Yield= 86%. Yellow needles, mp = 203-205 °C. ^1^H-NMR [(CD_3_)_2_CO] δ = 3.27 (s, 3H, NCH_3_); 3.95 (s, 3H, OCH_3_); 6.64 (s, 1H, C=CH); 6.92 (d, 1H, *J* = 8.7 Hz, H-5', Ar); 7.31 (d, 1H, *J* = 5.8 Hz, H-6', Ar); 7.32 (s, 1H, H-2', Ar); 8.25 (br s, 1H, OH); 10.84 (br s, 1H, NH). ^13^C-NMR [(CD_3_)_2_CO] δ = 26.70 (NCH_3_); 55.62 (OCH_3_); 113.33 (C-2); 113.51 (C=CH); 115.61 (C-5); 124.13 (C-6); 124.70 (C=CH); 124.84 (C-1); 147.82 (C-3); 148.53 (C-4); 16404 (C=O); 178.92 (C=S). 178.84 (C=S). HRMS, *m/z* found: 264.0552 (calculated for C_12_H_12_N_2_O_3_S, M^+^. requires: 264.0569).

*(5Z)-5-(2,3-Dihydro-1,4-benzodioxan-6-ylmethylene)-1-methyl-2-thioxoimidazolidin-4-one* (**4e**): *Method A*: The product **4e** was prepared from **1** (650 mg, 5 mmol), propylamine (0.8 mL, 10 mmol, 2 equiv.) and 1,4-benzodioxan-6-carboxaldehyde (**2e**, 1.540 g, 5 mmol) with a reaction of 60 min. Yield= 98%.

*Method B*: The product **4e** was prepared from 1 (1 g, 6.57 mmol) and *N*-[2,3-dihydro-1,4-benzo-dioxan-6-yl)methylene]propan-1-amine (**3e**, 1.12 mL, 13.16 mmol, 2 equiv.) with a reaction time 60 min. Yield= 92%.

Yellow needles, mp > 260 °C. ^1^H-NMR [(CD_3_)_2_SO] δ = 3.83 (s, 3H, OCH_3_); 4.29 (t, 2H, *J* = 6.8 Hz, OCH_2_CH_2_O); 4.30 (t, 2H, *J* = 6.8 Hz, OCH_2_CH_2_O); 6.34 (s, 1H, C=CH); 6.78 (d, 1H, *J* = 8.1 Hz, H-5', Ar); 7.06 (d, 1H, *J* = 8.3 Hz, H-6', Ar); 7.10 (s, 1H, H-2', Ar); 7.82 (br s, 1H, NH). ^13^C-NMR [(CD_3_)_2_CO] δ = 56.20 (OCH_3_); 63.81 (OCH_2_CH_2_O); 64.44 (OCH_2_CH_2_O); 110.12 (C=CH); 113.61 (C-2); 116.23 (C-5); 124.04 (C-6); 124.62 (C-1); 126.04 (C=CH); 148.21 (C-3); 148.42 (C-4); 166.53 (C=O); 175,.90 (C=S). HRMS, *m/z* found: 276.0642 (calculated for C_13_H_12_N_2_O_3_S, M^+^. requires: 276.0569).

### 3.3. General Procedure for the Synthesis of Compounds ***6*** by S-Alkylation of 5-Arylmethylene-1-methyl-2-thioxo imidazolin-4-ones ***4(a-g)*** with Halogeno Compounds ***5***

In a 10 mL two-necked round-bottomed flask, equipped with a magnetic stirrer and reflux condenser, a mixture of *(5Z)* 5-arylmethylene-1-methyl-2-thioxo-imidazolin-4-one **4** (10 mmoles), halogeno compound R-X **5** (1-1.5 equiv.), potassium carbonate (0.618 g, 5 mmoles, 0.5 equiv.) and, eventually potassium iodide KI (1.67 g, 10 mmoles) if X = Cl, Br for **5**, in dry acetonitrile, was stirred vigorously at the appropriate reaction temperature (60-81 °C) for 19-51 hours. After heating was completed, the reaction mixture was allowed to cool down to room temperature; the solvent and the volatile components were eliminated by rotary evaporation under reduced pressure. The crude residue was dissolved in ethyl acetate (20 mL), the contents were filtered through a sintered glass disc; the solution was recovered, and the solvent was removed under reduced pressure. The expected compound **6** was purified by recrystallization in EtOH. The precipitated product **6** was filtered off and further dried under high vacuum (10^−2^ Torr) at 30 °C for 1 hour, which gave the desired *(5Z)-* 2-alkylthio-5-arylmethylene-1-methyl-1,5-dihydro-imidazol-4-ones **6** as yellow solids. The pure compounds **6** were characterized by ^1^H-, ^13^C-NMR and HRMS.

*(5Z)-5-(1,3-Benzodioxol-5-ylmethylene)-2-ethylthio-1-methyl-1,5-dihydro-4H-imidazol-4-one* (**6a**): The product **6a** was prepared from *(5Z)-*5-(1,3-benzodioxol-5-ylmethylene)-1-methyl-2-thioxo-imidazolidin-4-one (**4a**, 2.34 g, 13.2 mmol), ethyl iodide (**5a**, 1.45 mL, 15.9 mmol, 1.5 equiv.) and potassium carbonate (816 mg, 6.61 mmol, 0.5 equiv.) in acetonitrile (40 mL) with a reaction time of 51 hours at 60 °C according to the general procedure. Yield= 60%. Yellow needles, mp > 260 °C. ^1^H- NMR [(CD_3_)_2_SO] δ = 1.50 (t, 3H, *J* = 7.4 Hz, CH_2_CH_3_); 3.12 (s, 3H, NCH_3_); 3.33 (q, 2H, *J* = 7.4 Hz, SCH_2_); 6.00 (s, 2H, OCH_2_O); 6.82 (d, 1H, *J* = 8.1 Hz, H-5', Ar); 6.84 (s, 1H, C=CH); 7.36 (dd, 1H, *J* = 8.1, 1.0 Hz, H-6', Ar); 8.03 (s, 1H, H-2', Ar). ^13^C-NMR [(CD_3_)_2_CO] δ = 14.71 (CH_2_CH_3_); 25.63 (SCH_2_); 26.92 (NCH_3_); 101.82 (OCH_2_O); 108.84 (C-5'); 111.20 (C-2'); 124.04 (C=CH); 128.83 (C-6'); 129.51 (C-1'); 137.52 (C=CH, C-5); 148.31 (C-4'); 149.50 (C-3'); 164.11 (C=N, C-2); 170,3 (C=O, C-4). HRMS, *m/z* found: 290.0730 (calculated for C_14_H_14_N_2_O_3_S, M^+^. requires: 290.0725).

*(5Z)**-**2-(Allylthio)-5-[(1,3-benzodioxol-5-yl)methylene)]-1-methyl-1,5-dihydro-4H-imidazol-4-one* (**6b**): The product **6b** was prepared from **4a** (500 mg, 1.91 mmol), potassium iodide (348 mg, 2.10 mmol, 1.1 equiv.), 3-bromopropene (**5b**, 348 mg, 0,25 mL, 2.10 mmol, 1.5 equiv.) and potassium carbonate (132 mg, 0.95 mmol, 0.5 equiv.) in acetonitrile (6 mL) with a reaction time of 48 hours at 66 °C according to the general procedure. Yield= 62%. Yellow needles, mp= 154-156 °C. ^1^H-NMR [(CD_3_)_2_SO] δ = 3.17 (s, 3H, NCH_3_); 4.02 (d, 2H, *J* = 7.1 Hz, SCH_2_CH=CH_2_); 5.15 (d, 1H, *J* = 10.2 Hz, SCH_2_CH=CH_2_); 5.40 (d, 1H, *J* = 17.2 Hz, SCH_2_CH=CH**_2_**); 6.00 (s, 2H, OCH_2_O); 6.10 (m, 1H, SCH_2_CH=CH_2_); 6.82 (d, 1H, *J* = 8.1 Hz, H-5', Ar); 6,83 (s, 1H, C=CH-Ar); 7.37 (dd, 1H, *J* = 8.1, 1 Hz, H-6', Ar); 8.05 (s, 1H, H-2', Ar). ^13^C-NMR [(CD_3_)_2_CO] δ = 26.51 (NCH_3_); 33.32 (SCH_2_); 101.80 (OCH_2_O); 108.52 (C-5'); 110.81 (C-2'); 119.51 (CH=CH_2_); 124.10 (C=CH-Ar); 128.04 (C-6'); 129.03 (C-1'); 132.01 (CH=CH_2_); 136.91 (C=CH, C-5); 148.02 (C-4'); 149.21 (C-3'); 163.04 (C=N, C-2); 169.91 (C=O, C-4). HRMS, *m/z* found: 302.0723 (calculated for C_15_H_14_N_2_O_3_S, M^+^. requires: 302.0725).

*(5Z)-5-[(1,3-Benzodioxol-5-yl)methylene]-1-methyl-2-propylthio-1,5-dihydro-4H-imidazol-4-one* (**6c**) : The product **6c** was prepared from **4a** (500 mg, 1.91 mmol), potassium iodide (348 mg, 2.10 mmol, 1.5 equiv.), 1-bromopropane (**5c**, 258 mg, 0,19 mL, 2.10 mmol, 1.5 equiv.) and potassium carbonate (132 mg, 0.95 mmol, 0.5 equiv.) in acetonitrile (6 mL) with a reaction time of 24 hours at 65 °C according to the general procedure. Yield= 65%. Yellow needles, mp= 102-104 °C. ^1^H-NMR [(CD_3_)_2_SO] δ = 1.11 (t, 3H, *J* = 7.4 Hz, CH_2_CH**_3_**); 1.89 (sext, 2H, *J* = 7.3 Hz, CH_2_CH_3_); 3.14 (s, 3H, NCH_3_); 3.31 (t, 2H, *J* = 7.2 Hz, SCH_2_); 6.04 (s, 2H, OCH_2_O); 6.82 (d, 1H, *J* = 8.1 Hz, H-5', Ar); 6.84 (s, 1H, C=CH); 7.36 (dd, 1H, *J* = 8.2, 1.4 Hz, H-6', Ar); 8.05 (d, 1H, *J* = 1.3 Hz, H-2', Ar). ^13^C-NMR [(CD_3_)_2_CO] δ = 13.51 (CH_2_CH_3_); 22.52 (CH_2_CH_3_); 26.50 (NCH_3_); 32.61 (SCH_2_); 101.41 (OCH_2_O); 108.40 (C-5'); 110.82 (C-2'); 123,.53 (C=CH); 128.04 (C-6'); 129.21 (C-1'); 137.12 (C=CH, C-5); 148.04 (C-4'); 149.03 (C-3'); 163.81 (C=N, C-2); 169.91 (C=O, C-4). HRMS, *m/z* found: 304.0861 (calculated for C_15_H_16_N_2_O_3_S, M^+^. requires: 304.0882).

*{[(5Z)-5-(1,3-Benzodioxol-5-ylmethylene)-1-methyl-4-oxo-4,5-dihydro-1H-imidazol-2-yl]thio} aceto-nitrile* (**6d**): The product **6c** was prepared from **4a** (500 mg, 1.91 mmol), chloroacetonitrile (**5d**, 76 mg, 63 μL, 1.0 mmol, 1 equiv.) and potassium carbonate (168 mg, 1.0 mmol, 0.5 equiv.) in acetonitrile (5 mL) with a reaction time of 19 hours at 81 °C according to the general procedure. Yield= 66%. Yellow needles, mp = 211-213 °C. ^1^H-NMR [(CD_3_)_2_SO] δ = 3.09 (s, 3H, NCH_3_); 4,47 (s, 1H, SCH_2_); 6.11 (s, 2H, OCH_2_O); 6.95 (s, 1H, C=CH); 7.00 (d, 1H, *J* = 8.1 Hz, H-5', Ar); 7,64 (dd, 1H, *J* = 8.1, 1.1 Hz, H-6', Ar); 8.11 (d, 1H, *J* = 1.2 Hz, H-2', Ar). ^13^C-NMR [(CD_3_)_2_CO] δ = 16.41 (SCH_2_); 26.84 (NCH_3_); 102.14 (OCH_2_O); 109.03 (C-5); 110.81 (C-2); 117.34 (CN); 124.71 (C=CH); 128.70(C-1'); 129.02 (C-6'); 136.51 (C=CH, C-5); 148.20 (C-4'); 149.71 (C-3'); 161.92 (C=N, C-2); 169.04 (C=O, C-4). HRMS, *m/z* found: 301.0507 (calculated for C_14_H_11_N_3_O_3_S, M^+^. requires: 301.0521).

*Ethyl {[(5Z)-5-(1,3-benzodioxol-5-ylmethylene)-1-methyl-4-oxo-4,5-dihydro-1H-imidazol-2-yl]thio}- acetate* (**6e**): The product **6e** was prepared from **4a** (200 mg, 0.78 mmol), potassium iodide (193 mg, 1.16 mmol, 1.5 equiv.), ethyl bromoacetate (**5e**, 192 mg, 1.16 mmol, 1.1 equiv.) and potassium carbonate (54 mg, 0.34 mmol, 0.5 equiv.) in acetonitrile (3 mL) with a reaction time of 14 hours at 60 °C according to the general procedure. Yield = 68%. Yellow needles, mp = 204-208 °C. ^1^H-NMR [(CD_3_)_2_SO] δ = 3.14 (s, 3H, NCH_3_); 1.17 (t, 3H, *J* = 7.4 Hz, CH_2_CH_3_); 2.49 (q, 2H, *J* = 7.3 Hz, CH_2_CH_3_); 4.36 (s, 2H, SCH_2_); 6.13 (s, 2H, OCH_2_O); 7,13 (d, 1H, *J* = 8.1 Hz, H-5', Ar); 7.21 (s, 1H, *J* = 1.3 Hz, C=CH); 7.74 (s, 1H, H-2', Ar); 8.13 (dd, 1H, *J* = 8.1, 1.5 Hz, H-6', Ar). ^13^C-NMR [(CD_3_)_2_CO] δ = 26.91 (NCH_3_); 14.22 (CH_2_CH_3_); 35.52 (CH**_2_**CH_3_); 62.11 (SCH_2_); 102.71 (OCH_2_O); 109.63 (C-5'); 109.81 (C-2'); 123.80 (C=CH); 127.43 (C-6'); 127.62 (C-1'); 136.44 (C=CH, C-5); 148.70 (C-4'); 150.43 (C-3'); 167.54 (C=N, C-2); 179.12 (C=O, C-4); 190.83 (C=O). HRMS, *m/z* found: 348.0741 (calculated for C_16_H_16_N_2_O_5_S, M^+^. requires: 348.0780).

*Ethyl {[(5Z)-5-(3,4-dimethoxybenzylidene)-1-methyl-4-oxo-4,5-dihydro-1H-imidazol-2-yl]thio}acetate* (**6f**): The product **6f** was prepared from *(5Z)-*5-(3,4-dimethoxybenzylidene)-1-methyl-2-thioxoimidazolidin-4-one (**4b**, 201 mg, 0.71 mmol), potassium iodide (177 mg, 1.07 mmol, 1.5 equiv.), ethyl bromoacetate (**5e**, 178 mg, 1.07 mmol, 1.5 equiv.) and potassium carbonate (49 mg, 0.36 mmol, 0.5 equiv.) in acetonitrile (3 mL) with a reaction time of 19 hours at 60 °C according to the general procedure. Yield= 62%. Yellow needles, mp = 252-254 °C. ^1^H-NMR [(CD_3_)_2_SO] δ = 3.14 (s, 3H, NCH_3_); 1.21 (t, 3H, *J* = 7.1 Hz, CH_2_CH_3_); 3.81 (s, 3H, OCH_3_); 3.82 (s, 3H, OCH_3_); 4.16 (q, 2H, *J* = 7.3 Hz, CH_2_CH_3_); 4.35 (s, 2H, SCH_2_); 7.11 (d, 1H, *J* = 8.1 Hz, H-5', Ar); 7.18 (s, 1H, C=CH) ; 7,22 (s, 1H, H-2', Ar); 7.78 (dd, 1H, *J* = 8.1, 1.4 Hz, H-6', Ar). ^13^C-NMR [(CD_3_)_2_CO] δ = 14.22 (CH_2_CH_3_); 26.91 (NCH_3_); 35.92 (CH_2_CH_3_); 55.91 (OCH_3_); 56.11 (OCH_3_); 62.13 (SCH_2_); 112.52 (C-5'); 113.83 (C-2'); 123,.33 (C=CH); 125.12 (C-6'); 126.10 (C-1'); 136.73 (C=CH, C-5); 149.42 (C-4'); 151.93 (C-3'); 167.53 (C=N, C-2); 179.14 (C=O, C-4); 190.51 (C=O). HRMS, *m/z* found: 364.1086 (calculated for C_17_H_20_N_2_O_5_S, M^+^. requires: 364.1093).

*(5Z)-5-(1,3-Benzodioxol-5-ylmethylene)-2-[(3-hydroxypropyl)thio]-1-methyl-1,5-dihydro-4H-imidaz-ol-4-one* (**6g**): The product **6g** was prepared from **4a** (150 mg, 0.57 mmol), potassium iodide (141 mg, 0.85 mmol, 1.5 equiv.), 3-bromopropanol (**5f**, 118 mg, 0.85 mmol, 1.5 equiv.) and potassium carbonate (39 mg, 0.28 mmol, 0.5 equiv.) in acetonitrile (4 mL) with a reaction time of 46 hours at 70 °C according to the general procedure. Yield= 42%. Yellow needles, mp= 256-258 °C. ^1^H-NMR [(CD_3_)_2_SO] δ = 1.98 (q, 2H, *J* = 7,4 Hz, CH_2_CH_2_OH); 2.03 (t, 2H, *J* = 7.3 Hz, SCH_2_); 3.31 (t, 2H, *J* = 7.2 Hz, CH_2_OH); 6.04 (s, 2H, OCH_2_O); 6.82 (d, 1H, *J* = 8,1 Hz, H-5', Ar); 6.84 (s, 1H, *J* = 1.3 Hz, C=CH); 7.36 (s, 1H, H-2', Ar); 8.13 (d, 1H, *J* = 8,2 Hz; H-6', Ar). ^13^C-NMR [(CD_3_)_2_CO] δ = 27.41 (NCH_3_); 29.52 (CH_2_CH_2_CH_2_OH); 31.07 (SCH_2_); 59.11 (CH_2_OH); 101.42 (OCH_2_O); 108.13 (C-5'); 109,8 (C-2); 118.73 (C=CH); 123.93 (C-6'); 126.83 (C-1'); 133.42 (C=CH, C-5); 148.72 (C-4'); 148.83 (C-3'); 168.52 (C=N, C-2); 177.91 (C=O, C-4).

## 4. Conclusions

In summary, new (*5Z*)-2-alkylthio-5-arylmethylene-1-methyl-1,5-dihydro-4*H*-imidazol-4-ones bearing two points of diversity have been developed according to a two step protocol. The first step involves a solvent-free Knoevenagel condensation under microwave irradiation that produced (*5Z*)-5-arylmethylene-2-thioimidazolidin-4-ones in good yields and high purity in a sterecontrolled fashion, followed by a chemoselective *S*-alkylation with retention of double bond configuration. Although a limited number of different and representative substituents on the 1-methyl-1,4-dihydro-4*H*-imidazol-4-one core are represented here, it's obvious that a much larger diversity can be easily achieved. Further studies are currently ongoing.
